# Dietary Supplementation with *Spirulina platensis* Modulates the Physiological Status and Bone Quality of Juvenile Ring-Necked Pheasants

**DOI:** 10.3390/ani16081127

**Published:** 2026-04-08

**Authors:** Sebastian Nowaczewski, Katarzyna Szkudelska, Joanna Składanowska-Baryza, Karolina Szulc, Agnieszka Ludwiczak, Krzysztof Kukulski, Daria Praska, Liliana Ciesielska, Sebastian Janiszewski, Bartosz Kierończyk

**Affiliations:** 1 Department of Animal Breeding and Product Quality Assessment, Faculty of Veterinary Medicine and Animal Science, Poznań University of Life Sciences, Szydłowska 50, 60-627 Poznań, Poland; sebastian.nowaczewski@up.poznan.pl (S.N.); joanna.skladanowska-baryza@up.poznan.pl (J.S.-B.); karolina.szulc@up.poznan.pl (K.S.); agnieszka.ludwiczak@up.poznan.pl (A.L.); 2 Department of Animal Physiology and Biochemistry, Faculty of Veterinary Medicine and Animal Science, Poznań University of Life Sciences, Wołyńska 35, 60-637 Poznań, Poland; katarzyna.szkudelska@up.poznan.pl; 3 OHZ Moszna, Powstańców Śląskich 51, 47-370 Zielina, Poland; 4 Department of Animal Nutrition, Faculty of Veterinary Medicine and Animal Science, Poznań University of Life Sciences, Wołyńska 33, 60-637 Poznań, Poland; dariapraska40@gmail.com (D.P.); cliliana040602@gmail.com (L.C.); 5 Avicare, Piekarska 4, 62-820 Stawiszyn, Poland; janiszewski.seb@gmail.com

**Keywords:** *Phasianus colchicus*, algae, performance, meat and bone quality, physiological status

## Abstract

This study evaluated the effects of dietary spirulina (*Spirulina platensis*) supplementation on the physiological status and bone quality of juvenile ring-necked pheasants. A total of 200 one-day-old chicks were randomly allocated to a control group fed a basal diet or to an experimental group fed a basal diet supplemented with 15 g of spirulina/kg feed and reared under identical conditions for 42 days. Growth performance, feed intake, the feed conversion ratio, selected carcass traits, meat and bone quality, and biochemical and hematological parameters were assessed. Pheasants receiving spirulina presented significantly greater body weights at four weeks of age. The experimental group also exhibited increased pH values in pectoral and thigh muscles and increased postmortem muscle temperature. Spirulina supplementation significantly improved tibia quality, as reflected by enhanced geometric, structural, and material bone properties. The birds fed spirulina presented increased plasma total cholesterol and low-density lipoprotein concentrations, whereas those in the control group exhibited increased malondialdehyde levels, indicating increased oxidative stress. In addition, spirulina increased the proportion of lymphocytes and reduced both the proportion and absolute number of monocytes. In conclusion, spirulina supplementation increased tibia bone strength, reduced oxidative stress, and had immunomodulatory effects on juvenile pheasants.

## Simple Summary

This study examined the effects of adding spirulina, a blue–green alga, to the diet of juvenile ring-necked pheasants. The birds were fed either a standard diet without additives or a diet supplemented with spirulina. Growth performance, meat and bone quality, and selected blood indicators related to physiological conditions and the immune response were evaluated. Pheasants receiving spirulina were slightly heavier at four weeks of age and presented greater leg bone strength. Some meat quality traits, including muscle acidity and temperature after slaughter, were affected. Spirulina supplementation reduced blood markers of oxidative stress and altered white blood cell profiles by increasing lymphocyte counts and decreasing monocyte counts. Birds fed spirulina also presented increased blood cholesterol levels, which may be related to changes in fat metabolism. Overall, spirulina did not markedly improve growth or most meat quality traits, but it increased bone strength, reduced oxidative stress, and may support immune function. These results suggest that spirulina may be a valuable dietary supplement for improving the health and resilience of young pheasants.

## 1. Introduction

The ring-necked pheasant (*Phasianus colchicus*) is gaining increasing importance across various sectors of the economy [1]. It is a popular and attractive game species widely utilized throughout Europe, and its meat is highly valued for its exceptional nutritional and sensory qualities [2]. Research has shown that pheasant breast and thigh muscles contain higher protein levels, lower fat contents, and significantly greater concentrations of most amino acids than broiler meat does [3,4]. Additionally, ring-necked pheasants can serve as valuable model organisms for identifying environmental factors that influence fluctuations in animal population numbers [5].

The pheasant is a species native to Asia. To survive in the natural environments of most countries where it is introduced, its populations must be continuously supplemented with birds from farm-based breeding programs. Under these conditions, pheasants are typically kept in a semi-intensive system—aviaries—where they are exposed to various unfavorable external factors, such as variable and often harsh weather conditions, as well as numerous pathogenic microorganisms. Individuals intended for release into the wild should be healthy and display appropriate behavioral traits. These factors significantly impact the post-release survival rate of pheasants and their long-term persistence on hunting grounds. It should be noted, however, that within the European Union, farm-reared pheasants are not only used for release into the wild but may also be kept for meat production, slaughtered, and marketed through retail chains, hotels, and restaurants. The meat of these birds is becoming increasingly popular and valued due to its nutritional properties, as discussed above.

Pheasants are typically released into their natural environment at 10–14 weeks of age. Therefore, during the rearing period and preparation for introduction, particular attention should be given to appropriate housing conditions, nutrition, and the management of stress. Unfortunately, studies have shown that young pheasants reared under intensive farming conditions are susceptible to numerous health issues. For example, pheasants aged 21–28 days are vulnerable to various diseases, including enteritis caused by caliciviruses, which resulted in a 25% mortality rate in a study conducted by Toffan et al. [6]. Common infections, such as colibacillosis, salmonellosis, and coccidiosis, as well as parasitic infestations such as syngamosis, are exacerbated by inadequate husbandry practices. In addition, poor housing conditions contribute to elevated stress levels and the occurrence of cannibalism, both of which adversely affect survival rates [7].

Consequently, various strategies are continuously being explored to improve pheasant health and reduce mortality, particularly among young birds reared in aviaries. In this context, a range of feed additives, including phytogenic compounds, are being investigated for their potential to increase the biochemical and physiological status of birds. Such improvements can contribute to better production performance and, ultimately, more favorable economic outcomes [8,9,10,11].

Spirulina (*Spirulina platensis*) is a species of blue–green algae recognized for its exceptional nutritional profile and health-promoting properties. It is rich in protein, essential vitamins (particularly B-group vitamins), minerals such as iron and calcium, and potent antioxidants. Its antioxidant activity helps mitigate oxidative stress, making it a valuable addition to animal diets. Numerous studies conducted on poultry, particularly broiler chickens, have confirmed the effectiveness of spirulina as a feed ingredient with multiple benefits. With a protein content ranging from 55% to 70%, along with essential amino acids, vitamins, and fatty acids, spirulina contributes to improved growth performance, immune response, and meat quality [12,13]. Research also indicates that spirulina can reduce the feed conversion ratio (FCR) and promote weight gain, especially when it is included in diets at levels less than 10% [14]. Other results showed that birds fed a diet supplemented with 15 g/kg *S. platensis* achieved superior body weights, body weight gain, and feed conversion ratios compared to the control [15]. Furthermore, the antioxidant properties of spirulina and its ability to influence the gut microbiota are key in supporting overall poultry health [13,16]. Moreover, the addition of spirulina to broiler diets (5 g/kg feed) increased erythrocyte and leukocyte counts and hematocrit and hemoglobin concentrations [17]. Comprehensive metabolic analysis revealed a significant increase in antioxidant capacity in birds fed spirulina diets, suggesting a possible decrease in oxidative stress [18]. Research on broiler chickens indicated that supplementation with 0.05% spirulina partially reduced the harmful effects of aflatoxin on body weight gain and the mass of the thymus and spleen. Adding microalgae to poultry feed may also significantly and positively influence bone parameters, such as the breaking strength [19], displacement at yield load in tibia bone, as well as ultimate load in femur [20]. Some studies on Japanese quail have shown that dietary supplementation with *S. platensis* at levels of 5–15 g/kg resulted in a linear improvement in growth performance, carcass yield, physiological condition, and immune function [21]. Based on the abovementioned benefits, from a practical perspective, the inclusion of *S. platensis* in diets for young pheasants may reduce variation in final body weight, improve feed efficiency, and enhance economic returns (meat production). Additionally, it may support bird health, particularly immunomodulatory effects, which could be beneficial in the context of releasing birds into the environment.

Despite its proven benefits in other poultry species, the impact of spirulina supplementation on the health and production parameters of young pheasants during the critical pre-release window (10–14 weeks of age) remains largely uninvestigated in the scientific literature. Therefore, the present study aimed to evaluate the impact of the inclusion of *Spirulina platensis* in young pheasant diets on growth performance, feed utilization, mortality, hematological and biochemical blood parameters, gastrointestinal tract morphology, slaughter and carcass characteristics, meat quality, and selected bone parameters related to strength. The authors hypothesize that the administration of *Spirulina platensis* in pheasant diets may improve growth performance, selected bone quality traits, and meat quality and physiological responses due to the presence of specific bioactive compounds and the additional macro- and microelements provided.

## 2. Materials and Methods

### 2.1. Birds and Housing

The experiment was conducted at a pheasant farm belonging to the Polish Hunting Association in Moszna, Poland (50°25′46.9″ N, 17°45′55.1″ E). The research material consisted of day-old chicks from a one-year-old breeding flock maintained at the same farm. After hatching, 200 chicks were randomly selected, marked (leg band), and weighed. The birds were then divided into two groups and reared under similar environmental conditions from day 0 to day 42. During the first three weeks, the pheasants were housed indoors in pens with a floor area of 18.5 m^2^, which were bedded with rye straw litter. On the first day post-hatching, the living area for the pheasants was maintained at 35.0 °C. During the rearing period, the ambient temperature was gradually reduced by approximately 1 °C per day. From days 8 to 14, the temperature ranged between 26 and 28 °C, and from days 15 to 21, it was maintained at 21–25 °C. Infrared heaters provided both heat and lighting. Relative humidity in the living area was kept at 55–60% until day 21. From the fourth week onward, the birds had access to restricted outdoor runs measuring 5.8 m × 20 m. During the study period, the average regional temperatures ranged from 27 °C to a minimum of 4 °C.

The experimental design included two dietary treatments. The control group (CON) received a basal diet without spirulina addition, whereas the experimental group (SP) received the same basal diet enriched with spirulina powder at a dose of 15 g/kg of feed added on top. Each group was subdivided into five replicate pens, with 20 leg-banded pheasants per pen. Since the trial was conducted on a commercial farm, the experimental birds were housed within a larger population of 450 individuals per pen to fully utilize the available rearing space.

The composition and nutritive value of the experimental diets are shown in Table 1 and Table 2. Additionally, the chemical composition of the experimental factor is presented in Table 3. Young pheasants had ad libitum access to feed and water throughout the entire experimental period. The basal diet was formulated according to the NRC guidelines [22] and was administered to birds aged 1–28 days (starter) and those aged 29–42 days (grower). The feed was produced according to ISO 9001:2015 procedures [23]. All the feed ingredients were mixed and pelleted (0.5 mm diameter) at 60 °C. The following feed additives were provided in the vitamin–mineral premix: phytase (500 FTU/kg), 1.4-beta-xylanase (250 FXU/kg), and coccidiostat lasalocid (120 mg/kg). Each batch of feed was stored in sealed 25 kg bags in a cool, dry place until used in feeding the birds.

### 2.2. Data and Sample Collection

The body weights (BWs) of the birds were measured weekly from day 1 to day 42 of rearing to calculate body weight gain (BWG). The average feed intake (AFI) and feed conversion ratio (FCR) were calculated for the entire rearing period. Mortality was monitored throughout the experiment at the pen level. Notably, BW and BWG were recorded only for the leg-banded birds, whereas the remaining performance parameters were calculated on the basis of the total number of pheasants in each pen (pen defined as experimental unit, *n* = 5). At the end of the trial (42 d), fifteen leg-banded male birds per treatment (*n* = 15; three randomly chosen birds per pen) were chosen for sampling. The birds were manually stunned by a percussive blow to the head and then sacrificed by cervical dislocation, followed by decapitation and exsanguination, and subsequently eviscerated, in accordance with Directive 2010/63/EU (Annex IV). Blood samples were collected immediately after decapitation into 2 mL tubes without anticoagulant. Serum was obtained by centrifugation (Micro 220 R, Hettich, Tuttlingen, Germany) at 1000× *g* and 8 °C for 10 min. The serum was carefully aspirated using a pipette, transferred into clean sample tubes, and stored at −20 °C for further biochemical analyses. Whole blood samples were separately collected into tubes (BD Vacutainer^®^, Becton Dickinson, Franklin Lakes, NJ, USA) containing an anticoagulant (heparin) for further hematological analysis. The birds were not subjected to feed restriction prior to blood sampling.

During evisceration, selected segments of the gastrointestinal tract (GIT) were rinsed in sterile water, drained, and weighed. Additionally, internal organs were collected for calculation of their weights relative to BW (% BW) via a laboratory balance (LPC-523i, VWR International, Leuven, Belgium; accurate to ±0.001 g) and their lengths relative to BW (cm/kg BW) via a linear scale accurate to 1 mm. The following empty GIT segments and internal organs were sampled: the duodenum, jejunum, ileum, ceca, gizzard, heart, liver, pancreas, spleen, and bursa of Fabricius. The jejunum was defined as the section beginning at the end of the duodenum and ending at Meckel’s diverticulum, whereas the ileum was considered the segment extending from Meckel’s diverticulum to the ileocecal junction. Next, the breast muscles from slaughtered birds (*n* = 15) were separated for further analyses. Additionally, the right tibia bone was collected from these birds (*n* = 15), cleaned of soft tissue remnants, weighed, and frozen (−20 °C) until analysis.

### 2.3. Carcass Characteristics

Carcass weights, including those of the neck and feet, were determined by weighing the entire slaughtered bird both before and after evisceration. The carcasses were subsequently dissected into breasts, leg quarters, drumsticks, and thighs, which were then weighed via a laboratory balance (NVL5101, OHAUS, Parsippany, NJ, USA; accurate to ±0.5 g). Carcass yield was calculated as the ratio of carcass weight to body weight at slaughter. The yield of individual parts (breast, leg quarters, drumsticks, and thighs) was expressed as the ratio of each part’s weight to the carcass weight. The giblets yield, comprising the heart, cleaned and emptied gizzards, and liver, was calculated as a percentage of giblets weight relative to carcass weight.

### 2.4. Meat Quality

Directly after slaughter, the left and right pectoralis major and thigh muscles were cut from each carcass. The muscles were weighed, exposed to 15- or 45-min blooming at room temperature (20–22 °C), and then stored at 3–6 °C. The pH and temperature were measured by inserting a glass-calomel electrode (Lo 406-M6-DXK-S7/25, Mettler Toledo, Columbus, OH, USA) connected to a pH meter (type 1140, Mettler Toledo, Columbus, OH, USA) into the right pectoralis major and thigh muscles. Two measurements per sample were taken at the following times postmortem: 15 min, 45 min, and 24 h. The mean value was used for further statistical analyses.

Additionally, color measurements were recorded on the surface of the meat after 15 and 45 min of blooming, and 24 h postmortem at three different positions on the exposed surface of each muscle (six measurements per bird), and the mean value was used for further statistical analyses. The CM 700d spectrophotometer was calibrated on a white and black calibration board (Konica Minolta, Amsterdam, The Netherlands) and had the following settings: illuminant D65, 10° observer, and aperture size, 8 mm diameter. Color measurements were made via the CIELab system, and the lightness (L*), redness (a*), and yellowness (b*) were recorded. The C* (Chroma) and h° (hue) values were calculated via spectrophotometer software. Color measurements were repeated at the following time points: 15 min, 45 min, and 24 h postmortem. All physiological measurements (pH, temperature, and color) were performed under standardized and identical conditions for all samples. The natural drip was measured via the EZ-Drip Loss method [24]. One 2.0 cm steak (approximately Ø 25 mm) was cut across the fiber direction of each left breast muscle 48 h post-mortem. The weight of each EZ-Drip Loss container was recorded first, followed by the weight of the container with the meat sample placed in the vertical fiber direction. The containers were sealed tightly and stored in a metal holder at 4 °C for 24 h. After that, the samples were removed from the containers, and the containers of meat juice were reweighed.

The cooking loss was measured right of the pectoralis major according to the modified method of [25]. The muscles were weighed and vacuum-packed in polyethylene sous vide bags (67 g/m^2^, density; ≤4.0 g/m^2^, oxygen permeability; ≤65 cm^3^/m^2^·day·bar, water vapor permeability). A thermocouple was inserted in the center of the additional sample (not used for data acquisition). The samples were placed in a water bath preheated to 76 °C and kept there until an internal temperature of 72 °C was reached (as measured in the additional sample). The samples were removed from the water bath, immediately immersed in an ice bath, transferred from vacuum bags to plastic bags, and placed in a refrigerator at 2–4 °C. The samples were then reweighed after 12 h.

The shear force measurements were performed using a Warner Bratzler (WB) V-shaped blunt blade attached to the TA.XT Plus Texture Analyzer (Stable MicroSystems, Warrington, UK) equipped with a 50 kg load cell (test speed: 2 mm/s; distance: 20 mm; trigger force: 0.049 N). The analysis was conducted on samples previously used for cooking loss determination and stored under chilled conditions (2–4 °C) for 12 h after cooking. Prior to analysis, samples were equilibrated to room temperature, and two samples (1.0 × 1.0 cm) were cut from each muscle parallel to the muscle fibers orientation.

Based on the recorded force–deformation curves, the following parameters were calculated using Exponent software (version 6.2, Stable Micro Systems, Warrington, UK): maximum shear force and shear energy. Additionally, the slopes of the force–deformation curve were determined over defined deformation intervals (0–10% and 20–80%). These parameters describe the resistance of muscle tissue to deformation at different stages of the shearing process. It should be noted that these values do not represent intrinsic material properties due to the complex and non-uniform stress distribution inherent to the Warner–Bratzler test. Therefore, these parameters are interpreted as apparent mechanical properties (apparent modulus), expressed as the slope of the force–deformation curve.

The use of force–deformation slopes as descriptors of mechanical resistance in muscle tissue is consistent with established approaches in food biomechanics, where such parameters are treated as apparent mechanical properties rather than intrinsic material constants [26,27].

### 2.5. Bone Quality

Bones wrapped in sterile saline-soaked gauze and placed in plastic bags were thawed overnight at 5 °C. Subsequently, prior to measurements, the samples were allowed to equilibrate until they reached room temperature for further analyses. Morphometric measurements of the tibia (*n* = 15) were taken in the anteroposterior and lateromedial directions at three levels: the proximal extremity, mid-diaphysis, and distal extremity. Measurements were performed via an electronic caliper with an accuracy of ±0.01 mm, according to the method described by Trela et al. [28]. The following parameters were recorded prior to biomechanical testing: tibia weight relative to BW (g/kg BW), bone length (mm), bone width (mm) at the aforementioned levels, and the Seedor index (mg/mm). A three-point bending test was subsequently performed via an Instron 34SC-2 testing machine (Instron GmbH, Darmstadt, Germany) equipped with a 2 kN load cell and operated with BlueHill Universal software (v. 4.55, Instron GmbH, Darmstadt, Germany). The test was carried out at a constant crosshead speed of 10 mm/min until bone fracture. The span length (distance between supports) was set individually at 40% of the total bone length. After the bones were fractured, internal diameters in the anteroposterior and lateromedial directions were measured (in the breaking place) to determine the following geometric properties: cross-sectional area (mm^2^), mean relative wall thickness, cortical index (%), cross-sectional moment of inertia (mm^4^), and radius of gyration (mm). Next, on the basis of the load–displacement curves, the following structural properties were determined: yield load (N), ultimate load (N), elastic energy (mJ), energy to fracture (mJ), and stiffness (N/mm). In terms of material properties, the following parameters were calculated: Young’s modulus of elasticity (GPa), yield strain, ultimate strain, bending moment (N·m), yield stress (MPa), and ultimate stress (MPa) according to Muszyński et al. [29].

### 2.6. Biochemical and Hematological Indices

Hematological analyses were performed via a Sysmex XN-VET analyzer (Sysmex Europe GmbH, Hamburg, Germany), which focused on red blood cell indices and total leukocyte count (without differentiation into specific leukocyte fractions). Total leukocyte counts were additionally verified via the manual chamber method. Blood smears were prepared and stained via the Pappenheim method [30], after which 100 consecutive leukocytes were examined microscopically and classified into granulocytes (heterophils, eosinophils, and basophils) and agranulocytes (lymphocytes and monocytes) to determine their percentage distribution. Blood biochemical parameters, including total protein, albumin, creatinine, triglycerides, total cholesterol, high-density lipoprotein (HDL), low-density lipoprotein (LDL)*,* glucose, uric acid, calcium, and phosphorus (all expressed in mg/dL), were measured via commercial diagnostic kits (Pointe Scientific, Inc., Canton Township, MI, USA) according to the manufacturer’s instructions. The malondialdehyde (MDA) concentration (nmol/M) was determined via the thiobarbituric acid (TBA) colorimetric method with a dedicated assay kit (Elabscience Bionovation Inc., Houston, TX, USA) following the manufacturer’s protocol. The activities of alanine aminotransferase (ALT; IU/L) and lactate dehydrogenase (LDH; IU/L) were also measured via Pointe Scientific assay kits in accordance with the manufacturer’s instructions.

### 2.7. Statistical Analyses

The experiment followed a completely randomized design. For growth performance analysis, the pen was considered the experimental unit (*n* = 5). In contrast, the individual bird (three randomly chosen pheasants per pen, *n* = 15) served as the experimental unit for morphometric measurements of selected organs, physicochemical traits of meat, bone quality assessments, and blood indices. Statistical analyses were performed via RStudio (v. 2025.05.1+513; Posit, PBC, Boston, MA, USA). Data normality was assessed via the Shapiro–Wilk test. Differences between treatment groups were evaluated via two-tailed Student’s *t* test for normally distributed data (significance threshold: *p* < 0.05). For variables with nonnormal distributions, the Mann–Whitney *U* test was applied at the same significance level. A trend toward significance was considered at *p* < 0.10.

## 3. Results

### 3.1. Growth Performance Parameters

Analysis of body weight gains and final body weights of the pheasants from week 1 to week 6 revealed no significant differences (*p* > 0.05) between the control and experimental groups (Table 4). The exception was observed at week 4, when pheasants receiving spirulina supplementation had significantly greater BW (by 25 g, *p* = 0.032). A tendency toward greater BW was also noted in week 3. No significant differences (*p* > 0.05) were found between the groups regarding FI, FCR, or mortality rate during the entire rearing period (Table 4).

### 3.2. Selected Internal Organ Measurements

The pheasant groups did not differ significantly in terms of the length or weight of individual GIT segments or selected internal organs (Table 5).

### 3.3. Carcass Traits and Meat Quality

No significant differences in slaughter traits were detected between the control group and pheasants fed the spirulina-supplemented diet (Table 6).

For the pectoralis major muscle, the only significant difference was observed in the L* parameter (Table 7), with lighter-colored meat found in the spirulina-fed group. Moreover, trends toward lower values of the C* parameter (15 min post-mortem) and b* and h° (24 h postmortem) were observed in the experimental pheasant group. Unlike the pectoral muscle, the thigh muscle of pheasants receiving spirulina-supplemented feed had a significantly higher pH (15 min and 24 h postmortem) (Table 8). Moreover, these birds presented a 2.1-point lower L* value in the thigh muscle (24 h postmortem; *p* = 0.048) and a 0.46-point higher a* value (*p* = 0.045).

### 3.4. Selected Bone Strength Indices

The addition of spirulina to the pheasant diets significantly (*p* < 0.05) affected bone quality, particularly with respect to selected geometrical, structural, and material parameters of the tibia bone (Table 9). A significant reduction in the external diameter of the bone in the anteroposterior plane (by 0.45 mm) was observed, which consequently decreased the cross-sectional area of the tibia (by 1.53 mm^2^) and the mean relative wall thickness in the experimental group. Additionally, spirulina supplementation led to a significant reduction in the tibia’s cross-sectional moment of inertia (mm^4^) and radius of gyration (mm). Furthermore, structural bone properties were influenced by spirulina, as evidenced by an increase in elastic energy (*p* = 0.023). The material properties also increased, as indicated by higher values of the Young’s modulus of elasticity (*p* < 0.001), yield stress (*p* < 0.001), and ultimate stress (*p* < 0.001).

### 3.5. Blood Biochemical Parameters and Hematological Indices

Pheasants receiving the spirulina-supplemented diet presented increased levels of total cholesterol and LDL in the plasma (Table 10). The differences compared with those in the control group were 13.0 mg/dL and 3.8 mg/dL, respectively, and were statistically significant. Conversely, the control group presented a significantly greater MDA concentration, exceeding that of the spirulina group by 0.61 nmol/mL. No significant differences in red blood cell parameters (RBC, HGB, and HCT) were detected between the groups. However, the leukogram analyses revealed a greater proportion of lymphocytes (by 12.08%) and a lower proportion and absolute count of monocytes (by 8.51% and 0.37 × 10^9^/L, respectively) in the spirulina-supplemented group (Table 11).

## 4. Discussion

Although the studied groups of pheasants did not differ in terms of average weekly weight gains, a tendency toward higher body weight, as well as a significantly greater value of this trait, was observed in the 3rd and 4th weeks of age, respectively, in birds receiving spirulina in their diet. These results are consistent with those of previous studies on poultry, indicating that spirulina increases nutrient absorption and feed efficiency because of its high protein and microelement contents [31,32]. In an experiment conducted on broiler chickens, the addition of spirulina to the feed (10 g/kg) significantly affected both weight gain and final body weight [33]. The differences between the experimental and control groups were 139 g and 127.8 g, respectively. These benefits may be due to the high protein levels of spirulina, the supply of essential amino acids, and the availability of bioactive substances that increase nutrient uptake and support gut health. Young pheasants exhibit a high growth potential during the first 4–5 weeks of life, which corresponds to their peak protein requirements. During this critical period, even modest improvements in nutrient availability, such as those provided by algal supplementation, may temporarily accelerate growth. However, as the birds age beyond this stage, their natural growth rate slows, and the relative effects of dietary supplements may decrease if the basal diet already fulfills their nutritional needs. Moreover, as pheasants approach physiological maturity, growth dynamics tend to stabilize, and compensatory growth or plateauing can occur, resulting in a convergence of body weights across different dietary treatments. This pattern explains why the early body-weight advantage observed at 4 weeks was no longer evident in later measurements. The authors also reported significant differences in FI and the FCR, favoring the group of chickens fed spirulina-supplemented feed. The present study revealed no differences in the FI, FCR, or mortality of pheasants up to 6 weeks of age. Similarly, Bonos et al. [16] did not observe any significant effects of the addition of spirulina to broiler diets on final BW (day 42), FCR, or mortality. The researchers analyzed two spirulina doses (5 and 10 g/kg of feed). However, its inclusion may not play such a critical role because Shinde et al. [34] reported significant differences between the control group and the experimental groups, in which broiler chickens received only 400, 600, and 800 mg of spirulina per 1 kg of diet. These differences concerned BWG, FI, and feed efficiency, with improved values observed in the experimental groups. It should be noted that an important limitation of the present study was the inability to determine the sex of the birds at the beginning of the experiment, as sexual dimorphism is not recognizable at this stage. Therefore, the results obtained in this trial do not exclude a potential effect of bird sex on the measured growth performance. Further studies are needed to continue the evaluation of pheasants fed diets with *Spirulina platensis* over a longer period with consideration of sex differences. Another limitation of the present study is related to the calculation of growth performance parameters, namely FI and FCR, which were determined at the pen level based on the entire population (mean of 450 birds per pen). Due to the experiment being conducted under field (commercial) conditions, it was not possible to use smaller replicate pens or separate individual birds to obtain more precise measurements. Additionally, a weakness of the trial is that the number of sampled birds exceeded the number of replicate pens. This may introduce pseudoreplication and should therefore be taken into account when interpreting the results. Although individual birds were used for downstream analyses, the experimental unit for performance-related traits remained the pen, which may influence the robustness of the statistical inference.

Spirulina is known to be responsible for the variation in broiler chickens’ meat quality attributes. One of the significant meat quality indicators is the pH value and its changes from slaughter (referred to as the initial pH, measured 15 min post-mortem in poultry) to stabilization 24–48 h post-mortem, referred to as the ultimate pH [35]. This parameter is not only used to detect myopathy but also to define the shelf-life of meat. In pheasants, the pH_15min_ should range from 5.74–5.94 in the breast muscle and from 6.47–6.68 in the thigh muscles [36].

Like forage-based diets, spirulina is rich in pigments, especially carotenoids. Thus, supplementation of this alga in the diet significantly changes the color of the meat [18,32]. We observed an effect of spirulina in the thigh muscles, which increased redness and yellowness. Costa et al. [37] and Spinola et al. [18] noted increased yellowness in broiler chicken meat due to spirulina supplementation. Interestingly, Altmann et al. [38] reported a more pronounced pigmentation effect in broiler chickens when a significant proportion of soy protein (50%) was substituted with spirulina, suggesting a dose-dependent response related to substitution level and feeding time. In contrast, in our study on pheasants, game birds, which are not intensively selected for fast growth, such as broilers, presented more subtle color changes, primarily in thigh muscles, which are richer in oxidative fibers and are more likely to accumulate pigments such as carotenoids. The limited response in terms of breast muscle coloration may also reflect lower pigment deposition due to differences in muscle metabolism and blood perfusion. Furthermore, the slaughter age (42 days) may have been too early to fully express the pigment accumulation effects, especially in species such as pheasants, which reach physiological maturity much later than broilers do. Additionally, spirulina supplementation had a minimal effect on breast meat texture, as evidenced by the similar Warner–Bratzler shear force and shear energy values between the control and spirulina-supplemented groups (*p* > 0.05). Similarly, no differences were observed in the apparent modulus (force–deformation slopes), indicating comparable resistance of muscle tissue to deformation between the experimental groups. These results align with those of Spínola et al. [18], who reported that moderate addition of spirulina to broiler diets had a neutral to slightly positive effect on meat tenderness, whereas higher levels offered no additional benefit. Similarly, Pestana et al. [39] reported that spirulina-enriched diets, especially when combined with enzyme supplementation, influenced certain meat quality traits without significantly altering textural properties.

While relationships between muscle fiber characteristics and meat quality have been extensively described in poultry [40], extrapolation of these findings to pheasants should be approached with caution. Available studies on pheasants have primarily focused on general meat quality traits and compositional differences rather than detailed muscle histochemistry [2,3,4]. Moreover, pheasants, as non-selected game birds, differ substantially from intensively selected broiler chickens in growth rate, activity, and muscle development, which may influence postmortem metabolism and physicochemical properties of the meat [3]. In general, in avian physiology, breast muscles are typically associated with more glycolytic metabolism, whereas leg muscles exhibit more oxidative characteristics [41]. These metabolic differences are closely related to postmortem biochemical processes, particularly glycogen depletion and the rate of pH decline. Muscles with a more glycolytic profile typically exhibit a faster decline in pH due to higher glycogen content and more intensive postmortem glycolysis, whereas more oxidative muscles tend to show a slower pH decline [3].

However, the extent to which this general pattern applies to pheasants remains insufficiently documented, and direct evidence regarding muscle fiber distribution in this species is limited. Therefore, in the absence of species-specific histochemical or biochemical data, the present results can only suggest potential differences in muscle metabolic properties rather than confirm specific fiber-type distribution. Consequently, the observed variation in pH decline and color parameters is more appropriately interpreted as reflecting differences in postmortem metabolism without direct attribution to muscle fiber composition. Further studies incorporating muscle fiber typing and enzymatic profiling in pheasants are required to elucidate the underlying mechanisms.

The addition of *S. platensis* to the diet of mammals positively affects tibial bone growth and strength, as evidenced by increased yield load, maximum load, stiffness, and work-to-fracture [42]. Moreover, it has been shown to suppress synovial inflammation and inhibit osteoclast differentiation through oxidative stress regulation, ultimately restoring bone homeostasis and alleviating erosion [43]. Similarly, spirulina supplementation has positive effects on bone quality in birds. In broiler chickens, both the tibia and femur exhibited greater displacement at the yield load and ultimate load, respectively [20]. Furthermore, Oh et al. [19] demonstrated that even a minimal inclusion level of microalgae (1 and 2 mg/kg) in the diets of Pekin ducks can enhance tibial physicochemical properties by increasing the bone breaking strength, although the bone length and crude ash content remain unaffected.

There are real scientific indications that farm-raised pheasants may have a weakened leg bone structure and other anatomical limitations, which reduce their chances of survival after being released into the wild [44,45]. Therefore, the leg health of pheasants kept in aviaries appears to be a key factor in achieving high survival rates once the birds are released into the natural environment. As expected, the results of the present study are consistent with previous findings [19,20], confirming that inclusion of microalgae can positively influence tibial quality, as demonstrated by increased displacement at yield load and greater breaking strength. These results clearly indicate an increased capacity for energy absorption under load (elastic energy), enhanced bone tissue stiffness (as shown by Young’s modulus of elasticity), and improved tibial strength, which is supported by increased resistance of the tibia to deformation and fracture (yield and ultimate stress). Simultaneously, tibial geometrical indices, such as the cross-sectional area, mean relative wall thickness, cross-sectional moment of inertia, and radius of gyration, resulted in decreased values. This outcome may be explained by a compensatory effect, which is not related to an increase in bone dimensions but rather to structural remodeling of the bone. However, on the basis of blood biochemical parameters (Ca and P levels), it may be suggested that bone mineralization remained unaffected. This observation is consistent with the findings of El-Hady et al. [46], who reported no significant effect of spirulina supplementation (at 3% or 6%) in broiler diets on calcium and phosphorus concentrations in the blood or tibia, despite its administration, which was greater than that reported in the present study. This finding is in line with the nonsignificant changes in the Seedor index, which is used to indirectly indicate the mineralization of bone. Thus, the authors hypothesize that the high concentration of vitamin C in *S. platensis*, along with its role as a co-factor in collagen synthesis and crosslink formation, may contribute to maintaining bone structure and elasticity, which plays a pivotal role in bone mechanical properties [42]. Moreover, the phycocyanin and *β*-carotene contents of this microalga exhibit anti-inflammatory properties and reduce oxidative stress [46], which may also positively affect overall bone health. This finding aligns with the significantly lower MDA concentration observed in the plasma of pheasants receiving spirulina supplementation. However, this proposed mechanism requires further investigation, particularly regarding the composition of the intestinal microecosystem and the bioavailability of macro- and microelements in the GIT of birds. Nevertheless, the observed improvements in tibial bone strength appear to be valuable factors in better preparing well-conditioned pheasants for successful release into the natural environment.

The analyses revealed that spirulina did not cause significant changes in the general health or metabolism of the animals. It did not affect the function of the liver or kidneys, as indicated by the unchanged concentrations of total protein and albumin in the blood, no changes in the activities of ALT and LDH, or in the concentrations of creatinine and uric acid. Spirulina is distinguished by its high protein content. However, in our experiment, the supplement did not cause any changes in the concentration of either total protein or albumin in the pheasants. The results of other studies also revealed that, in broilers after consuming this supplement, the concentration of protein in the blood does not change, especially the concentration of albumin [47], and very few studies have reported results indicating an increase in protein concentration [48,49]. Spirulina also did not interfere with the energy metabolism of the animals; the concentrations of the primary substrates used in these processes, glucose and triglycerides, remained within the range of physiological values.

An unexpected change observed in the experiment, especially in the context of reports from the scientific literature, was the significant increase in the concentration of total cholesterol in the blood of pheasants and cholesterol transported in LDL observed as a result of spirulina intake. Most of the results described by the researchers have focused on broilers and indicate that this alga reduces the concentrations of both parameters, as well as triglycerides, while increasing the concentration of cholesterol in the HDL fraction, and the effect is interpreted, as in humans, as health-promoting. However, it should be noted that such effects were observed in birds subjected to heat stress [50,51], and these parameters were restored to physiological values due to the presence of spirulina. Similar effects were observed in broilers in the production cycle, i.e., birds with a changed, more intensive metabolism, with a predominance of anabolic processes, additionally receiving much higher doses of spirulina—30 and 60 g per kg—in these animals, and spirulina also reduced the concentration of total lipids in the blood and cholesterol [52]. However, some reports indicate that broilers fed with 15% dietary inclusion of spirulina presented an increase in total serum lipids, including triglycerides, and total and LDL-cholesterol [30,39]. Notably, in our study, despite a significant increase in total cholesterol and cholesterol concentrations in the LDL fraction, these parameters remained within the range of physiological values. It can be assumed that the effect of spirulina was beneficial for young animals, probably compensating for the demand for cholesterol during intensive growth. The increase in cholesterol due to the effect of spirulina in these animals could have occurred because of an increase in lipid synthesis in the liver and/or transport to the tissues to provide an adequate supply of this compound for the building of the cell membrane and steroidogenesis.

A diet enriched with spirulina is also an important factor that benefits the body’s antioxidant defense mechanisms. The few studies on broilers thus far indicate that this factor increases the total antioxidant capacity of serum and TAC [53] and increases the activity of the following antioxidant enzymes in plasma: catalase, superoxide dismutase and glutathione peroxidase [32]. These changes are associated with a reduction in the level of the lipid oxidation marker MDA. In our study, we also observed a significant decrease in the level of MDA in the serum under the influence of spirulina in pheasants. Some researchers claim that reducing the oxidative potential may be beneficial for growing poultry, even though it may also improve the weight gain of animals.

It is well known that *Spirulina platensis* enhances immune responses by modulating both innate and adaptive immunity. Its bioactive compounds, such as phycocyanins and polysaccharides, increase macrophage phagocytic activity, stimulate T and B lymphocyte proliferation, and upregulate cytokine production (IL-2, IFN-γ), engaging immune pathways including TLR signaling [54,55,56]. In broilers, spirulina supplementation also shifted leukocyte profiles, decreasing heterophils and increasing lymphocytes, while micronutrients such as zinc further support cellular immunity [57,58,59,60]. Our studies also revealed a significant *(p* < 0.05) increase in the percentage of lymphocytes in the blood of young pheasants receiving spirulina in their diets. On the other hand, this study demonstrated a decrease in monocyte number and percentage in birds fed a diet supplemented with spirulina. Some insight into this phenomenon was shed by Qureshi et al. [61]. Although the total count of monocytes may remain relatively unchanged, their proportion within the white blood cell differential typically decreases owing to a substantial rise in lymphocyte numbers. This reflects a change in the balance of cell types rather than a true reduction in monocyte production.

## 5. Conclusions

Supplementation of *Spirulina platensis* (15 g/kg) did not clearly improve growth performance in young pheasants. However, it affects muscle color and postmortem meat characteristics, which should be assessed at the end of the standard rearing period (10–14 weeks) to confirm the results. Despite its ability to slightly reduce tibial geometric parameters, spirulina improved bone mechanical strength without compromising elasticity, indicating a potential benefit for skeletal durability. Additionally, *S. platensis* modulated certain physiological responses, though its effects on lipid metabolism require further investigation.

## Figures and Tables

**Table 1:** Composition of the experimental diet for pheasants fed from day 1 to 28 (g kg^−1^, as-fed basis).

Ingredients, g kg^−1^	Basal Diet
Soybean meal	430
Wheat	340
Maize	108
Hemoglobulin	22.0
Fishmeal	20.0
Soybean oil	11.5
Sunflower meal	10.0
Rapeseed cake	10.0
Dicalcium phosphate	20.0
Monocalcium phosphate	9.00
Limestone	3.80
Na_2_SO_4_	2.50
NaCl	1.70
^1^ Mineral-vitamin premix	11.5
Total	1000
Calculated chemical composition, g kg^−1^	
Metabolizable energy, MJ/kg	11.7
Crude protein	280
Crude fat	41.0
Crude fiber	33.5
Crude ash	72.0
Lysine	17.7
Methionine	5.80
Calcium	11.1
Total phosphorus	9.00

^1^ Provided the following per kilogram of vitamin–mineral premix: vitamin A, 10,000 IU; cholecalciferol, 3500 IU; vitamin E, 50 IU; Cu (CuSO_4_), 14 mg; Fe (FeSO_4_), 50 mg; Mn (MnO_2_), 70 mg; Zn (ZnSO_4_), 70 mg; I (CaI_2_O_6_), 1.0 mg; Se (Na_2_SeO_3_), 0.3 mg; BHT, 1.91 mg; BHA, 0.14 mg; propionic acid, 0.3 g; formic acid, 0.9 g; and sodium formate, 0.15 g.

**Table 2:** Composition of the experimental diet for pheasants fed from days 29–42 (g kg^−1^, as-fed basis).

Ingredients, g kg^−1^	Basal Diet
Wheat	340
Maize	211
Soybean meal	175
Rapeseed meal	70.0
Expanded soybean	65.0
Triticale	52.6
Hemoglobulin	24.0
Fishmeal	20.0
Soybean oil	2.50
Dicalcium phosphate	15.0
Monocalcium phosphate	5.20
Limestone	5.90
Na_2_SO_4_	1.20
NaCl	1.90
^1^ Mineral-vitamin premix	10.7
Total	1000
Calculated chemical composition, g kg^−1^	
Crude protein	220
Crude fat	35.5
Crude fiber	33.0
Crude ash	65.0
Lysine	13.1
Methionine	4.73
Calcium	10.1
Total phosphorus	8.60

^1^ Provided the following per kilogram of vitamin–mineral premix: vitamin A, 10,000 IU; cholecalciferol, 3500 IU; vitamin E, 50 IU; Cu (CuSO_4_), 14 mg; Fe (FeSO_4_), 50 mg; Mn (MnO_2_), 70 mg; Zn (ZnSO_4_), 70 mg; I (CaI_2_O_6_), 1.0 mg; Se (Na_2_SeO_3_), 0.3 mg; BHT, 1.91 mg; BHA, 0.14 mg; propionic acid, 0.3 g; formic acid, 0.9 g; and sodium formate, 0.15 g.

**Table 3:** Chemical composition of *Spirulina platensis*.

Item	Contents per 100 g *S. platensis*
Dry matter, g	90.9
Crude protein, g	58.1
Crude fat, g	6.56
Crude fiber, g	2.37
Crude ash, g	6.67
Nitrogen-free extract, g	17.2
Calcium, mg	488
Phosphorus, mg	1035
Sodium, mg	527
Potassium, mg	1634
Iron, mg	109
Zinc, mg	3.09
Isoleucine, g	4.26
Leucine, g	5.95
Valine, g	4.56
Lysine, g	3.44
Tryptophan, g	0.92
Phenylalanine, g	4.12
Methionine, g	1.60
Threonine, g	3.63

**Table 4:** Effect of spirulina supplementation in the diet of young pheasants on growth performance parameters.

Item	Treatments		^3^ SEM	*p*-Value
	^1^ CON	^2^ SP		
Body weight, g				
Initial weight	19.7	19.7	0.09	0.980
Week 1	32.1	35.8	1.14	0.150
Week 2	65.5	67.5	1.80	0.629
Week 3	111	133	7.7	0.057
Week 4	169 b	194 a	6.3	0.032
Week 5	236	241	5.5	0.713
Week 6	298	307	9.8	0.695
Body weight gain, g				
Week 1	12.3	15.9	1.08	0.128
Week 2	31.5	32.5	1.29	0.738
Week 3	50.3	61.7	4.23	0.114
Week 4	58.5	65.6	2.49	0.200
Week 5	55.5	57.1	1.06	0.494
Week 6	75.7	81.6	4.22	0.525
Feed intake, g				
Overall	0.986	0.972	0.0151	0.664
Feed conversion ratio, g:g				
Overall	1.62	1.56	0.049	0.574
Mortality rate, %				
Overall	2.17	1.85	0.184	0.439

^1^ CON, control group fed a basal diet; ^2^ SP, experimental treatment group fed a basal diet enriched with spirulina; ^3^ SEM, standard error of the mean. The means represent 3 randomly chosen birds per pen (*n* = 5). a,b Values within a row with different letters are significantly different (*p* < 0.05).

**Table 5:** Effect of spirulina supplementation in the diet of young pheasants on the selected gastrointestinal tract segments and internal organ weights (% body weight) and lengths (cm kg^−1^ body weight), d42.

Item	Treatments		^3^ SEM	*p*-Value
	^1^ CON	^2^ SP		
Length, cm kg^−1^ body weight				
Duodenum	39.2	38.4	1.00	0.700
Jejunum	88.8	86.5	1.79	0.528
Ileum	93.3	94.0	2.78	0.932
Cecum	43.0	43.9	1.67	0.811
Weight, % body weight				
Duodenum	1.36	1.44	0.048	0.165
Jejunum	1.51	1.47	0.047	0.817
Ileum	0.90	0.91	0.036	0.885
Cecum	0.81	0.89	0.044	0.387
Gizzard	2.38	2.30	0.092	0.931
Heart	0.50	0.51	0.008	0.816
Pancreas	0.34	0.35	0.011	0.882
Liver	2.43	2.51	0.060	0.520
Spleen	0.11	0.10	0.005	0.524
Bursa of Fabricius	0.17	0.19	0.010	0.355

^1^ CON, control group fed a basal diet; ^2^ SP, experimental treatment group fed a basal diet enriched with spirulina; ^3^ SEM, standard error of the mean. The means represent 3 randomly chosen birds per pen (*n* = 15).

**Table 6:** Effect of spirulina supplementation in the diet of young pheasants on the selected carcass traits, d42.

Item	Treatments		^3^ SEM	*p*-Value
	^1^ CON	^2^ SP		
Final body weight, g	430	424	9.7	0.771
Carcass weight, g	308	307	7.5	0.945
Carcass yield, %	71.6	72.4	0.39	0.344
Breast weight, g	29.0	28.6	0.75	0.783
Breast yield, %	18.9	18.6	0.32	0.696
Leg quarters, g	39.0	38.8	0.99	0.918
Leg quarters yield, %	25.4	25.2	0.20	0.722
Drumstick weight, g	19.6	19.3	0.53	0.736
Drumstick yield, %	12.8	12.5	0.15	0.435
Thigh’s weight, g	19.4	19.5	0.51	0.879
Thigh’s yield, %	12.6	12.7	0.15	0.773
Giblets weight, g	22.7	22.5	0.57	0.816
Giblets yield, %	7.44	7.33	0.186	0.843

^1^ CON, control group fed a basal diet; ^2^ SP, experimental treatment group fed a basal diet enriched with spirulina; ^3^ SEM, standard error of the mean. The means represent 3 randomly chosen birds per pen (*n* = 15).

**Table 7:** Effect of spirulina supplementation in the diet of young pheasants on the selected breast meat quality traits, d42.

Item	Treatments		^3^ SEM	*p*-Value
	^1^ CON	^2^ SP		
15 min post-mortem				
pH	6.08	6.10	0.030	0.677
Temp (°C)	33.6	29.6	2.41	0.908
L*	44.7 a	43.0 b	0.40	0.026
a*	0.019	0.177	0.1621	0.638
b*	7.30	6.77	0.166	0.117
C*	7.38	6.80	0.167	0.083
h°	90.0	88.5	1.32	0.589
45 min post-mortem				
pH	5.83	5.94	0.053	0.337
Temp (°C)	26.8	27.7	0.55	0.424
L*	47.1	45.1	0.74	0.168
a*	0.65	0.66	0.201	0.983
b*	7.18	7.25	0.295	0.898
C*	7.31	7.33	0.298	0.964
h°	84.6	85.0	1.58	0.893
24 h postmortem				
pH	5.70	5.72	0.020	0.750
Temp (°C)	12.5	12.7	0.40	0.977
L*	52.8	51.9	0.53	0.431
a*	0.50	0.81	0.127	0.224
b*	7.59	6.82	0.233	0.098
C*	7.66	6.89	0.236	0.106
h°	86.8	83.4	0.95	0.069
Physicochemical traits				
Drip loss, %	1.04	1.12	0.114	0.751
Cooking loss, %	13.0	12.1	0.49	0.378
Shear Force Max, N	17.8	19.0	1.46	0.932
Shear Energy, N·mm	161	169	10.5	0.887
Apparent modulus, 0–10%	0.656	0.661	0.0328	0.936
Apparent modulus, 20–80%	1.40	1.34	0.098	0.478

^1^ CON, control group fed a basal diet; ^2^ SP, experimental treatment group fed a basal diet enriched with spirulina; ^3^ SEM, standard error of the mean. The means represent 3 randomly chosen birds per pen (*n* = 15). a,b Values within a row with different letters are significantly different (*p* < 0.05).

**Table 8:** Effect of spirulina supplementation in the diet of young pheasants on the selected thigh muscle quality traits, d42.

Item	Treatments		^3^ SEM	*p*-Value
	^1^ CON	^2^ SP		
15 min post-mortem				
pH	6.31 b	6.49 a	0.042	0.030
Temp (°C)	35.0	35.3	0.35	0.725
L*	50.6	50.7	0.55	0.862
a*	1.73	1.70	0.204	0.671
b*	7.60	7.99	0.194	0.322
C*	7.93	8.27	0.184	0.364
h°	76.7	82.0	2.80	0.799
45 min post-mortem				
pH	6.23	6.17	0.038	0.392
Temp (°C)	25.9 b	27.7 a	0.49	0.039
L*	48.8	49.7	0.52	0.387
a*	1.40	1.54	0.234	0.769
b*	7.42	6.78	0.251	0.207
C*	7.67	7.16	0.240	0.301
h°	79.4	87.4	3.10	0.630
24 h postmortem				
pH	6.31 b	6.52 a	0.045	0.015
Temp (°C)	19.2	19.2	0.48	0.944
L*	51.5 a	49.4 b	0.53	0.048
a*	1.20 b	1.66 a	0.195	0.045
b*	5.87	6.12	0.196	0.291
C*	6.08	6.38	0.223	0.378
h°	79.6	74.8	1.47	0.109

^1^ CON, control group fed a basal diet; ^2^ SP, experimental treatment group fed a basal diet enriched with spirulina; ^3^ SEM, standard error of the mean. The means represent 3 randomly chosen birds per pen (*n* = 15). a,b Values within a row with different letters are significantly different (*p* < 0.05).

**Table 9:** Effect of spirulina supplementation in the diet of young pheasants on the selected geometrical, structural and material properties of tibia bone, d42.

Item	Treatments		^3^ SEM	*p*-Value
	^1^ CON	^2^ SP		
Tibia mass (g/kg ^4^ BW)	5.82	5.70	0.086	0.498
Seedor index (mg/mm)	31.0	30.3	0.66	0.615
Geometrical properties				
Length, mm	80.4	79.5	0.76	0.583
^5^ AP plane proximal width, mm	9.39	9.15	0.157	0.453
^6^ LM plane proximal width, mm	13.9	13.6	0.16	0.489
AP plane external diameter B, mm	4.29 a	3.84 b	0.081	0.004
AP plane internal diameter b, mm	2.58	2.49	0.042	0.288
LM plane external diameter H, mm	4.59	4.53	0.062	0.623
LM plane internal diameter h, mm	3.15	3.12	0.064	0.831
AP plane distal width, mm	6.80	6.68	0.084	0.476
LM plane distal width, mm	10.1	10.4	0.11	0.288
Cross-sectional area, mm^2^	9.06 a	7.53 b	0.287	0.005
Mean relative wall thickness, -	0.563 a	0.502 b	0.0175	0.038
Cortical index, %	31.5	31.2	0.92	0.859
Cross-sectional moment of inertia, mm^4^	15.5 a	10.3 b	0.95	0.005
Radius of gyration, mm	1.29 a	1.16 b	0.025	0.008
Structural properties				
Yield load, N	66.7	72.4	2.48	0.264
Ultimate load, N	96.0	92.8	3.52	0.661
Elastic energy, mJ	14.6 b	18.2 a	0.83	0.023
Energy to fracture, mJ	45.1	45.1	2.49	0.999
Stiffness, N/mm	158	151	5.1	0.545
Material properties				
Young modulus of elasticity, GPa	28.1 b	39.5 a	1.82	<0.001
Yield strain, -	0.011	0.012	0.0003	0.128
Ultimate strain, -	0.020	0.019	0.0006	0.828
Bending moment, N·mm	0.541	0.573	0.0224	0.486
Yield stress, MPa	77.8 b	109 a	4.5	<0.001
Ultimate stress, MPa	111 b	140 a	4.7	<0.001

^1^ CON, control group fed a basal diet; ^2^ SP, experimental treatment group fed a basal diet enriched with spirulina; ^3^ SEM, standard error of the mean; ^4^ BW, body weight; ^5^ AP, anteroposterior direction; ^6^ LM, lateromedial direction. The means represent 3 randomly chosen birds per pen (*n* = 15). a,b Values within a row with different letters are significantly different (*p* < 0.05).

**Table 10:** Effect of spirulina supplementation in the diet of young pheasants on the selected plasma biochemical parameters, d42.

Item	Treatments		^3^ SEM	*p*-Value
	^1^ CON	^2^ SP		
Total protein, mg/dL	2.95	2.92	0.039	0.708
Albumin, mg/dL	1.40	1.40	0.021	0.899
Creatinine, mg/dL	0.258	0.279	0.0240	0.182
^4^ ALT, IU/L	5.01	5.06	0.351	0.941
Triglycerides, mg/dL	93.4	85.8	3.87	0.332
Total cholesterol, mg/dL	114 b	127 a	2.7	0.019
^5^ HDL, mg/dL	86.3	91.3	2.48	0.316
^6^ LDL, mg/dL	18.0 b	21.8 a	0.90	0.037
^7^ MDA, nmol/mL	3.77 a	3.16 b	0.135	0.018
Glucose, mg/dL	358	353	5.4	0.718
^8^ LDH, IU/L	138	144	5.1	0.885
Uric acid, mg/dL	7.87	8.90	0.501	0.483
Calcium, mg/dL	9.10	8.94	0.125	0.877
Phosphorus, mg/dL	6.78	6.10	0.286	0.404

^1^ CON, control group fed a basal diet; ^2^ SP, experimental treatment group fed a basal diet enriched with spirulina; ^3^ SEM, standard error of the mean; ^4^ ALT, alanine aminotransferase; ^5^ HDL, high-density lipoprotein; ^6^ LDL, low-density lipoprotein; ^7^ MDA, malondialdehyde; ^8^ LDH, lactate dehydrogenase. The means represent 3 randomly chosen birds per pen (*n* = 15). a,b Values within a row with different letters are significantly different (*p* < 0.05).

**Table 11:** Effect of spirulina supplementation in the diet of young pheasants on the selected blood hematological traits, d42.

Item	Treatments		^3^ SEM	*p*-Value
	^1^ CON	^2^ SP		
^4^ WBC, ×10^9^/L	4.71	4.56	0.278	0.800
^5^ RBC, ×10^12^/L	2.45	2.56	0.051	0.284
^6^ HGB, g/L	75.30	76.36	1.735	0.770
^7^ HCT, l/L	0.32	0.33	0.007	0.798
Heterophils, %	21.10	18.54	2.425	0.612
Heterophils, ×10^9^/L	1.05	0.85	0.151	0.751
Eosinophils, %	0.10	0.09	0.065	1.000
Eosinophils, ×10^9^/L	0.003	0.003	0.002	1.000
Basophils, %	10.10	9.09	1.164	0.677
Basophils, ×10^9^/L	0.46	0.39	0.050	0.501
Lymphocytes, %	47.10 b	59.18 a	3.082	0.044
Lymphocytes, ×10^9^/L	2.21	2.73	0.205	0.202
Monocytes, %	21.60 a	13.09 b	1.845	0.019
Monocytes, ×10^9^/L	0.95 a	0.58 b	0.085	0.027
^8^ H:L	0.48	0.40	0.071	0.588

^1^ CON, control group fed a basal diet; ^2^ SP, experimental treatment group fed a basal diet enriched with spirulina; ^3^ SEM, standard error of the mean; ^4^ WBC, white blood cell; ^5^ RBC, red blood cell; ^6^ HGB, hemoglobin; ^7^ HCT, hematocrit; ^8^ H:L, heterophil–lymphocyte ratio. The means represent 3 randomly chosen birds per pen (*n* = 15). a,b Values within a row with different letters are significantly different (*p* < 0.05).

## Data Availability

The original contributions presented in the study are included in the article, and further inquiries can be directed to the corresponding author.
